# On-treatment measurements of circulating tumor DNA during FOLFOX therapy in patients with colorectal cancer

**DOI:** 10.1038/s41698-020-00134-3

**Published:** 2020-11-13

**Authors:** Tina Moser, Julie Waldispuehl-Geigl, Jelena Belic, Sabrina Weber, Qing Zhou, Samantha O. Hasenleithner, Ricarda Graf, Jasmin Alia Terzic, Florian Posch, Heinz Sill, Sigurd Lax, Karl Kashofer, Gerald Hoefler, Helmut Schoellnast, Ellen Heitzer, Jochen B. Geigl, Thomas Bauernhofer, Michael R. Speicher

**Affiliations:** 1 Institute of Human Genetics, Diagnostic and Research Center for Molecular BioMedicine, Medical University of Graz, Graz, Austria; 2 Department of Internal Medicine Graz, Division of Oncology, Medical University of Graz, Graz, Austria; 3 Department of Internal Medicine, Division of Hematology, Medical University of Graz, Graz, Austria; 4 Department of Pathology, General Hospital Graz II, Graz, Austria, and Johannes Kepler University Linz, Linz, Austria; 5 Institute of Pathology, Diagnostic and Research Center for Molecular BioMedicine, Medical University of Graz, Graz, Austria; 6 Department of Radiology, Division of General Radiology, Medical University of Graz, Graz, Austria; 7 BioTechMed-Graz, Graz, Austria; 8 Christian Doppler Laboratory for Liquid Biopsies for Early Detection of Cancer, Graz, Austria; 9 Present Address: Cancer Research UK Cambridge Institute, University of Cambridge, CB2 0RE, Cambridge, UK

**Keywords:** Biomarkers, Cancer

## Abstract

We addressed a significant unknown feature of circulating tumor DNA (ctDNA), i.e., how ctDNA levels change during chemotherapy, by serially monitoring ctDNA in patients with colorectal cancer during the 48-h application of FOLFOX. Surprisingly, we did not observe a spike in ctDNA as a sign of a responsive tumor, but instead ctDNA levels initially decreased and remained low in patients with stable disease or partial response. Our observations reveal further insights into cell destruction during chemotherapy with important implications for the management of patients.

## Introduction

Colorectal cancer (CRC) represents a major public health problem as one of the most frequent solid cancers^1^. Cell-free DNA (cfDNA), which contains circulating tumor DNA (ctDNA) in patients with cancer, is an emerging biomarker in precision oncology^2^, as ctDNA reflects CRC tumor burden^3^ and early ctDNA dynamics may predict the outcome of chemotherapy^4,5^. However, despite these promising prospects, many features of ctDNA as a biomarker remain unknown. The aim of our study was to address one of these unknowns, i.e., the on-treatment ctDNA effects during chemotherapy.

## Results

**Fig. 1:**
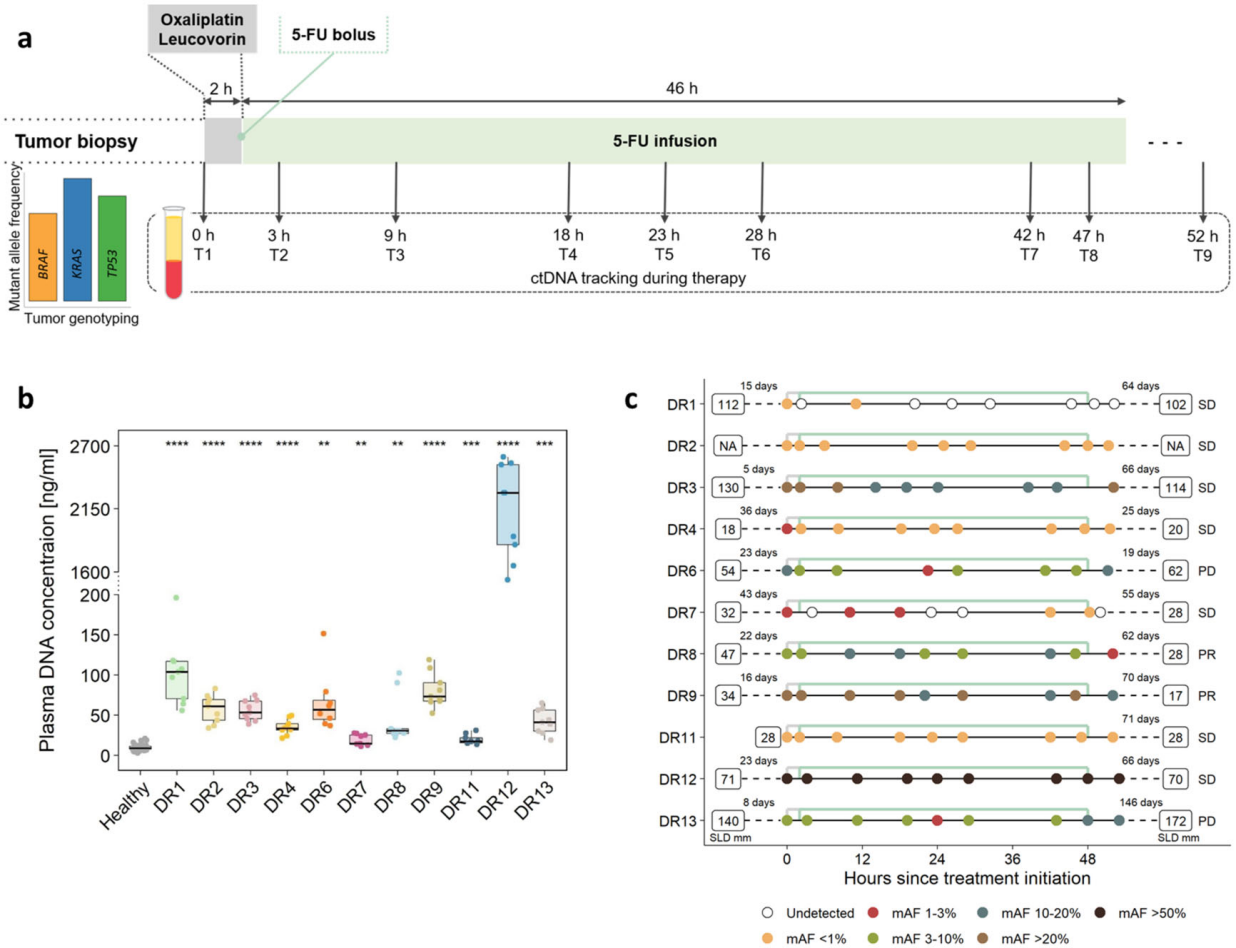
Study outline and plasma parameters. **a** Schematic overview of the FOLFOX regimen and the time points of blood collection. All tumor tissues were analyzed for mutations in *KRAS*, and in some cases, *BRAF* and *TP53* were sequenced as well. **b** Plasma DNA quantities of the patients and healthy controls (*n* = 60) (Student’s *t*-test; **p < 0.01, ***p < 0.001, ****p < 0.0001). All boxplots indicate the minimum and maximum value and median (center), and the interquartile range is shown by box and whiskers. **c** The median mutant allele frequencies (mAFs) for each plasma DNA analysis and the timing of each blood draw are displayed. Furthermore, the sum of longest diameters (SLD) as established by CT imaging for the selected target lesions according to RECIST are shown prior to (left side) and after (right side) completion of the FOLFOX cycle.

We studied a cohort of 13 patients (DR1-DR13) with the inclusion criteria of metastasized CRC and progressive disease under one of the most widely used chemotherapies, i.e., FOLFOX (FOLinic acid (leucovorin), Fluorouracil (5-FU), OXaliplatin; Supplementary Note 1 and Supplementary Note 2; Supplementary Table 1; Fig. 1a). FOLFOX is administered over a 48-h period and continuously destroys dividing cancer cells (ref. ^6^; Supplementary Note 1). In order to capture treatment-associated release of ctDNA, we used a tight blood collection schedule: The baseline (T1) and the last (T9) blood samples were drawn before and after FOLFOX treatment, respectively, whereas seven further blood samples (T2 to T8) were collected during administration (Fig. 1a) with the exception of DR6, from whom we received only six blood samples. Two patients, i.e., DR5 and DR10, were excluded due to withdrawn consent or treatment status.

For all patients, we established the *KRAS* mutation status from tumor tissues and in a subset, we additionally sequenced *TP53* and *BRAF* (Supplementary Table 1; Fig. 1a). Somatic mutations in these genes are prime trunk mutant alleles in CRC, which were shown to parallel clinical response^7^. We then started by analyzing all cfDNA samples for somatic copy number alterations (SCNAs)^8^ and determined the respective tumor fractions with the ichorCNA algorithm^9^ (Supplementary Fig. 1) (limit of detection (LOD) of 3% tumor fraction; Supplementary Note 3). In three cases (DR3, DR8, DR12) with higher colon-derived DNA proportions (≥10%), we used deep sequencing (LOD 1%)^10^ to study the fluctuation of the respective tumor-specific *KRAS* mutations. In all other cases, we employed ultra-sensitive methods comprising SiMSen-seq (Simple, multiplexed, PCR-based barcoding of DNA for sensitive mutation detection using sequencing)^11^ and the AVENIO ctDNA Targeted Kit, which covers 17 genes^12^ (LOD 0.1% each). The AVENIO kit was used to test for the presence of further mutations in selected plasma samples (DR2, DR4, DR9, and DR 13; Supplementary Note 3) and then to track in all plasma samples from DR4 five (2 mutations in *APC*, and one each in *KIT, TP53*, and *KRAS*) and from DR9 two mutations (*TP53* and *BRAF*). We employed SiMSen-seq to monitor the *KRAS* (DR1, DR6, DR7, DR11), *TP53* (DR13), or *KRAS* and *TP53* (DR2) mutations in all plasma samples. Extensive performance and consistency assessments demonstrated high reliability and robustness of the assays (Supplementary Note 3).

In agreement with previous reports^13^, we found that advanced CRC patients had significantly increased amounts of cfDNA per milliliter of plasma (median: 50.3 ng/ml; range: 11.0–2,602.8 ng/ml) compared to healthy controls (median: 8.8 ng/ml; range: 2.9–20.8 ng/ml) (Student’s *t*-test, *p* < 0.01–0.0001; Fig. 1b; Supplementary Note 3). One patient, DR12, had exceptionally increased plasma DNA concentrations (Fig. 1b; Supplementary Fig. 2).

**Fig. 2:**
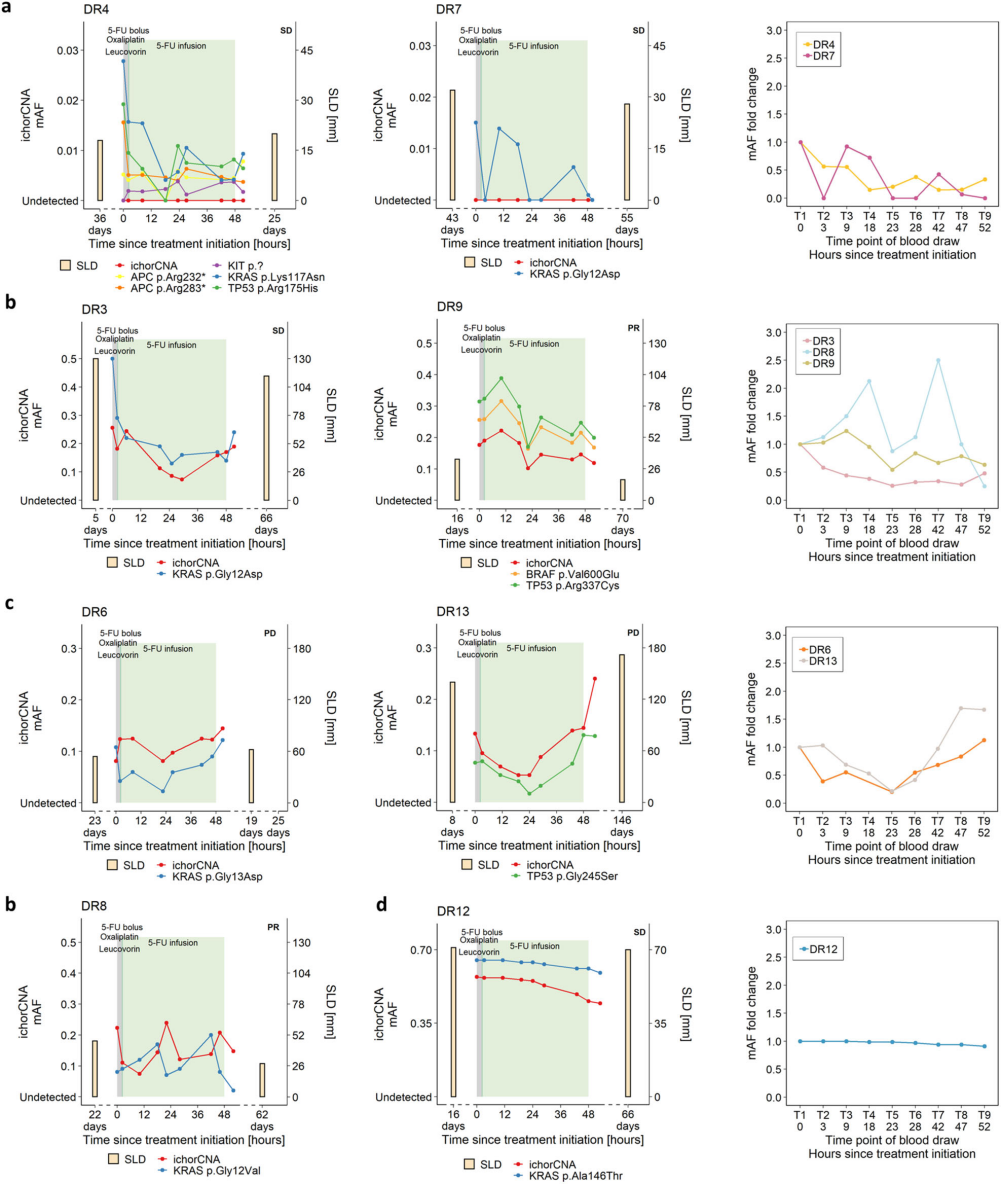
ctDNA level changes during FOLFOX administration. In each panel, the tumor fraction as established by ichorCNA is displayed in red and the respective mutations in various colors. Before and after the FOLFOX cycle, the sum of longest diameters (SLD) for the target lesions is displayed. Fold changes in plasma ctDNA mAFs are each displayed in the right panel. **a** ctDNA levels of two patients (DR4 and DR7) with low ctDNA mAFs. **b** Three patients (DR3, DR8, DR9) with ctDNA mAFs that decrease below baseline values during the FOLFOX cycle. For reasons of clarity, the results of DR8 are shown in the bottom row. **c** Two patients (DR6, DR13) whose mAFs increased during the last 24 h of the treatment cycle to values higher than baseline levels. **d** ichorCNA, *KRAS*, and SLDs of patient DR12.

In plasma from three patients (DR1, DR2, DR11), the ctDNA mutant allele frequencies (mAFs) were invariably at levels of 0.4% or less throughout the entire 48-h treatment cycle (Fig. 1c; Supplementary Fig. 3), confirming previous reports that there may be no correlation between the size of the target lesion (diameters of target lesions were 112 mm and 28 mm for patients DR1 and DR11, respectively; Supplementary Note 2) and sample mAF in plasma^2,14^. In five patients (DR4, DR7, DR3, DR8, DR9) (Fig. 1c), we observed a decrease of the ctDNA mAF within the first 18–23 h and after treatment (T9), mAFs were lower than at baseline (T1) (Fig. 2a–b; Supplementary Fig. 4). However, in the plasma of two patients (DR6, DR13), the initial decrease of mAFs within the first 23 h was followed by an increase of mAFs higher at T9 than at T1 (Fig. 2c; Supplementary Fig. 5). The mAF fold changes in patient DR12, who demonstrated remarkably elevated cfDNA (Fig. 1b) and the highest ctDNA mAFs in plasma, were consistent with an at best modest decrease of ctDNA (Fig. 1c and 2d). Compared to barcoding and deep sequencing, the orthogonal ichorCNA analyses provided established mAFs closely congruent to SCNA-derived mAFs (Pearson’s R = 0.98, *p* < 2.2 × 10^–16^) (Supplementary Fig. 6), which confirmed the high reliability of our analyses (Fig. 2b–d).

**Fig. 3:**
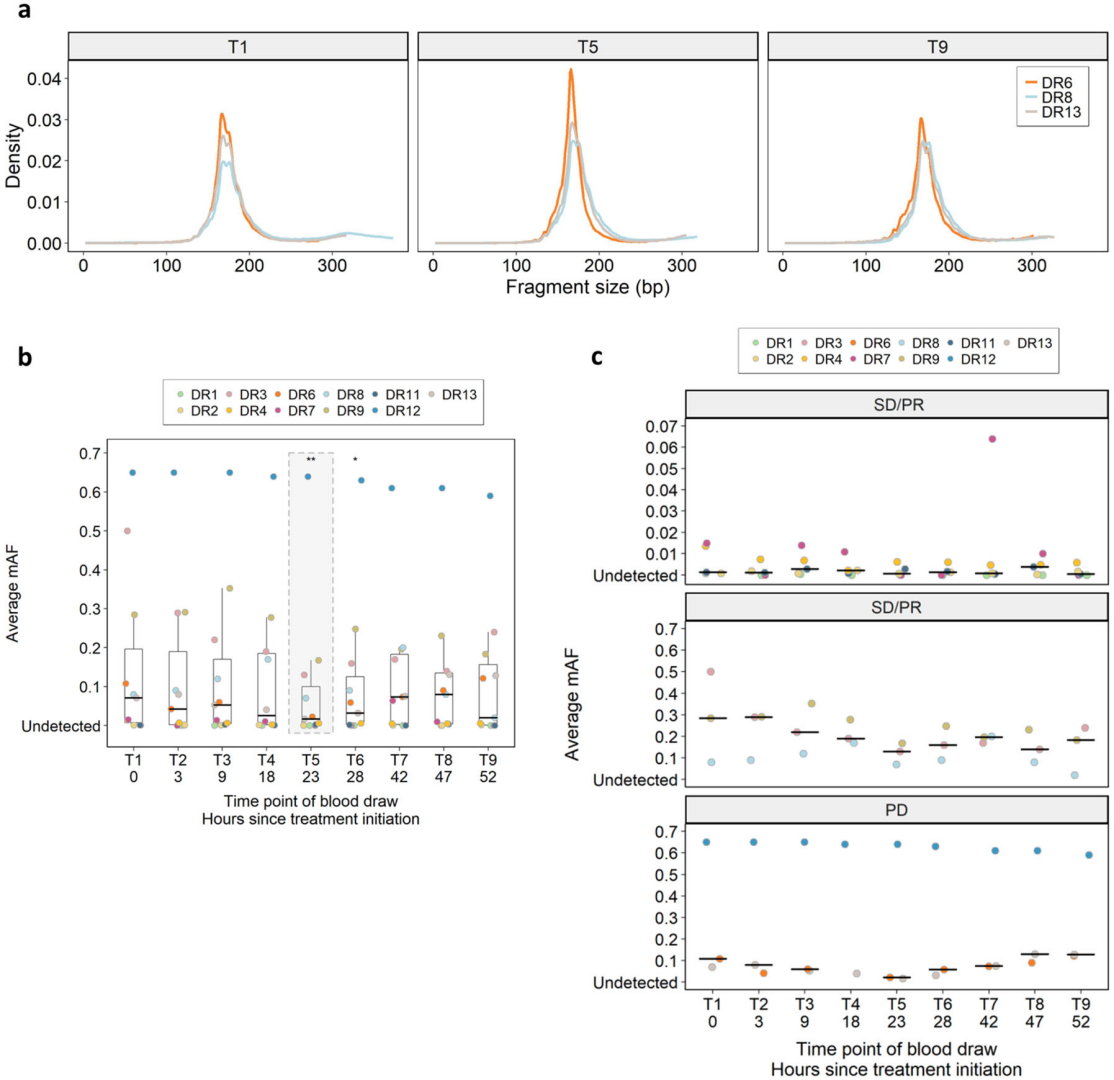
cfDNA size patterns and initial associations with treatment response. **a** cfDNA size profiles determined from paired-end sequencing data from patients DR6, DR8, and DR13 at time points T1, T5, and T9. **b** Serial comparison between baseline mAFs (T1) and mAFs at other time points (*p < 0.05, **p < 0.005, paired Wilcoxon test). The boxplots indicate the minimum and maximum value and median (center), and the interquartile range is shown by box and whiskers. **c** Patients with stable disease (SD) or partial response (PR) and very low ctDNA mAFs (DR1, DR2, DR4, DR7, and DR11) are shown in the upper panel and those with high ctDNA mAFs (DR3, DR8, and DR9) in the center panel. Patients with progressive disease (PD) (DR6, DR12, and DR13) are displayed in the lower panel.

We also addressed possible mechanisms of DNA release under therapy, such as apoptosis or necrosis^15,16^. Necrotic cells release high-molecular weight DNA^15^. However, using electrophoresis, we did not observe any evidence for the presence of such molecules (Supplementary Fig. 7). Instead, cfDNA fragmentation patterns were consistent with those previously associated with release from apoptotic cells, i.e., a modal value of DNA fragments lengths near 166 bp, which corresponds to the DNA wrapped around a nucleosome (~147 bp) plus a linker fragment (~20 bp)^17^. Importantly, this pattern did not change during therapy (Fig. 3a). As reported earlier^18,19^, we also observed that plasma DNA fragments from CRC patients were shorter than those of healthy individuals (*p* < 0.0001) (Supplementary Fig. 7). Taken together, the plasma DNA fragmentation patterns under FOLFOX therapy did not differ from those generally observed in patients with cancer^2,18,19^.

Finally, comparison of the baseline (T1) mAFs with the mAFs at all other time points revealed that the most significant mAF drop was at T5 (*p* < 0.005), which corresponds to about 23 h after start of treatment (Fig. 3b). When we applied RECIST (Response Evaluation Criteria in Solid Tumors) 1.1 criteria^20^ and compared patients with stable disease or partial response with those with progressive disease (Supplementary Note 4), we observed that in the stable disease/response group, the ctDNA mAFs remained at decreased levels at the time of our last blood collection, i.e., T9 (between T1 and T5: *p* = 0.03906; between T1 and T9: *p* = 0.01563) (Fig. 3c).

## Discussion

The main limitation of our study is the modest sample size, which can be attributed to the considerable logistical efforts and especially additional burden and discomfort imposed on the patients due to the tight blood drawing schedule requiring a great willingness to help on the part of the patient and their families. Furthermore, patients have to be treated as inpatients for the numerous blood draws, while FOLFOX is increasingly being administered on an outpatient basis. As a consequence, there is only a small number of other studies with a relative tight monitoring schedule and all of them have a limited patient number in common. In patients with non-small-cell lung cancer (NSCLC), one study analyzed daily kinetic changes of *EGFR* mutation levels in urine from nine patients^21^ and another study quantified *EGFR* and *KRAS* mutations in three patients over defined time periods, but only in one on a daily basis, i.e., every 24 h^22^. A further study analyzed pre- and postchemotherapy samples in five patients with metastatic prostate cancer within 1 h of chemotherapy infusion^23^.

However, to the best of our knowledge, no previous study conducted a comparable tight sampling schedule from peripheral blood during an intravenous chemotherapy as we did and this unique setting allowed us to make unexpected observations. The most surprising finding was that although drugs such as oxaliplatin or 5-FU are swiftly distributed throughout tissue and quickly destroy dividing cells throughout the entire 48-h period of administration (ref. ^6^; Supplementary Note 1), we did not observe a spike in ctDNA in any patient, which may have reflected a rapid release of tumor DNA into the circulation from responsive tumors. Instead, we invariably observed a pattern of ctDNA mAF decrease in all cases. Interestingly, that decreased ctDNA levels at the end of a therapy may indicate therapy response was also suggested in the abovementioned NSCLC and prostate cancer studies^22,23^. In our study, patients with progressive disease showed a trend towards a ctDNA mAF increase between T5 and T9 (Figs. 2c and 3c); however, the increase did not reach significant levels, most likely due to the low patient number.

In conclusion, further studies are clearly required before the clinical utility can be evaluated. For clinical applications the optimal timing of ctDNA level measurements, whether in the first hour posttreatment instead of at day 5 or day 7 or later still needs to be established. To this end, our work may initiate early dynamic therapy response modeling and contribute to the establishment of a real-time personalized treatment response assessment.

## Methods

### Blood/sample collection and processing

The study was approved by the Ethics Committee of the Medical University of Graz (approval number 26–288 ex 13/14 for the study part involving patients under FOLFOX treatment and 29–272 ex 16/17 for the collection and analysis of blood samples from healthy controls) conducted according to the Declaration of Helsinki. Written informed consent was obtained from all patients and healthy individuals, respectively.

For each patient, we collected plasma samples prior to initiation of FOLFOX treatment and during therapy for a total of nine plasma samples each (Fig. 1a). One exception was patient DR6, for whom we obtained only eight samples. Nine milliliters of blood were collected into BD Vacutainer^®^ EDTA tubes containing 10% NBF (BD Biosciences) or Streck tubes. Plasma was separated as described previously^8^ and stored at −80 °C prior to DNA isolation.

The healthy control group consisted of individuals between 20 and 29 years of age (mean 26 years). In addition, we conducted further comparisons with an age-matched control group from publicly available data^24^ (see Supplementary Note 3 for further details).

### cfDNA extraction and quantification

cfDNA extraction and quantification were done according to our experiences from a multicenter study and all details were described previously^25^. In brief, plasma DNA was extracted from 2 ml of plasma using the QIAamp Circulating Nucleic Acid Kit (QIAGEN) according to the manufacturer’s protocol, which included the addition of the same amount of carrier RNA to each sample. Extracted DNA was quantified by the Qubit dsDNA HS Assay Kit (Thermo Fisher Scientific) and stored at −20 °C before analysis.

### Tumor genotyping

Tumor tissue was available for all patients and the *KRAS* mutation status was assessed from the primary tumor (all patients except DR4) or metastatic sites (DR4). In addition, the primary tumors from patient DR7, DR9, and DR13 were examined for changes in *TP53* and/or *BRAF*. The Ion AmpliSeq Colon and Lung Cancer Panel v2 (Thermo Fisher Scientific), the Therascreen KRAS Pyro Kit (QIAGEN) or the Idylla KRAS and NRAS-BRAF-EGFR S492R Mutation Assays (Biocartis) were used to establish the mutation status in these genes directly from formalin-fixed paraffin-embedded (FFPE) tissue sections. Tumor genotyping was carried out at the Diagnostic and Research Institute of Pathology, Medical University of Graz and at the Department of Pathology, General Hospital Graz II, Graz, Austria.

For 9 of 11 patients (82%), specific mutations were available from the primary tissue, in one patient (DR4) only metastatic material was available for molecular profiling and in the tumor of one patient (DR13), no mutation was identified.

### Plasma-seq and ichorCNA analysis

For all samples, shallow whole-genome sequencing (sWGS), i.e., plasma-Seq^8,26^, was performed to establish genome-wide copy number alterations. Shotgun libraries were prepared from 5–10 ng plasma DNA using the TruSeq DNA Nano Sample Preparation Kit (Illumina, San Diego, CA, USA) as previously described^8,26^. Pooled libraries were quantified by qPCR and sequenced on the Illumina MiSeq or NextSeq platform using paired-end (2x75bp; *n* = 47 CRC samples) or single-end mode (1x150bp; *n* = 51 CRC samples). In addition, tumor fraction of each sWGS dataset was estimated using the previously published ichorCNA algorithm^9^. Samples with a tumor fraction below 0.03 were evaluated as ctDNA-negative, since this value was previously defined as the lower limit of sensitivity for detecting the presence of tumor DNA.

### AVENIO ctDNA targeted panel

For mutation profiling, we used the commercially available AVENIO ctDNA Targeted Kit, which covers 17 genes across 81 kb, including those in the U.S. National Comprehensive Cancer Network (NCCN) Guidelines, using hybrid capture targeted enrichment techniques (*ALK*, *APC*, *BRAF*, *BRCA1*, *BRCA2*, *DPYD*, *EGFR*, *ERBB2*, *KIT*, *KRAS*, *MET*, *NRAS*, *PDGFRA*, *RET*, *ROS1*, *TP53*, *UGT1A1*) (Roche). In brief, sequencing libraries were generated from 10–20 ng of plasma-derived DNA according to the manufacturer’s instructions. Libraries were quantified by qPCR and the quality was assessed using a Bioanalyzer High Sensitivity Kit (Agilent Technologies). Pooled libraries were sequenced on the Illumina NextSeq platform using a 2x150bp paired-end mode. On average, 15 million paired-end reads were obtained per sample (range 12.7–17.3 M). Sequencing data were analyzed using the AVENIO ctDNA Analysis Software (Roche) and variant calls were generated with a customized workflow using defined somatic variant filter settings. We excluded synonymous variants and variants present with >1% mutant allele frequency in population frequency databases (ExAC, gnomAD, 1000genomes).

### Simple, multiplexed, PCR-based barcoding of DNA for sensitive mutation detection using sequencing (SiMSen-seq)

For tracking mutations in plasma samples with low ctDNA levels, we employed a molecular barcoding approach, SiMSen-seq. First, target-specific primers were designed to capture each tumor’s selected mutations. For DR1, DR2, DR6, DR7 and DR11, various *KRAS* assays were designed (i.e., *KRAS* p.Ala146Thr, p.Gly12Asp, p.Gly13Asp). Moreover, for DR2, we used a 2-plex assay to track a *KRAS* mutation (p.Gly12Asp) and a *TP53* variant (p.Ser215Gly). For DR13, we monitored a *TP53* mutation (p.Gly245Ser) identified from AVENIO. Target-specific primer sequences were as follows: KRAS_1-F: TTTACCTCTATTGTTGGATCATATTCGTCCA and KRAS_1-R: GCCTGCTGAAAATGACTGAATATAAACTTGTG; KRAS-436-2-F: TTTCAGTGTTACTTACCTGTCTTGT and KRAS-436-2-R: GGCTCAGGACTTAGCAAGAAGT; TP53_7-F: CCTGGAGTCTTCCAGTGTGATG and TP53_7-R: GACTGTACCACCATCCACTACAAC; TP53_P223_1-F: CCTCCCAGAGACCCCAGTT and TP53_P223_1-R: GCGTGTGGAGTATTTGGATGAC.

SiMSen-seq libraries were generated from 20 ng of plasma DNA as described previously in detail^27^. In brief, this protocol involves an initial three PCR cycle step, in which template DNA was tagged and target regions were enriched using barcoded target-specific primers. During a second round of PCR, Illumina-specific adapters were attached to barcoded PCR products. PCR products were purified using the Agencourt AMPure XP system (Beckman Coulter, Inc). Libraries were quantified using the QIAseq Library Quant Assay (QIAGEN) and the library quality was assessed using the Bioanalyzer High Sensitivity DNA Kit (Agilent Technologies). Sequencing was performed on an Illumina MiSeq platform in single-end 150 bp mode, with a spike-in of 15% PhiX Control v3 (Illumina). Sequencing data generated by SiMSen-seq were analyzed using Debarcer (De-Barcoding and Error Correction). At least three reads with the same barcode (consensus 3) were required to form a valid barcode family.

### Establishment of the limit of detection for SiMSen-seq and the AVENIO assay

The performance of the assays was evaluated using the SeraCare reference material (Seraseq ctDNA v2), which harbors multiplexed variants in various genes at defined mutant allele frequencies (mAF). While the wild-type sample clearly showed negative results, both UMI-based methods were able to detect variants with a detection limit as low as 0.1% mAF. Therefore, the analytical sensitivity was set to 0.1% mAF.

### Deep sequencing

We conducted targeted deep sequencing for the *KRAS* p.Gly12Asp, p.Gly12Val and p.Ala146Thr mutations in all plasma samples from patients DR3, DR8 and DR12.

Target-specific primer sequences were as follows: KRAS_12_13_F: AGGCCTGCTGAAAATGACTG and KRAS_12_13_R: TTGTTGGATCATCTTCGTCCAC; KRAS_A146T_F: TGTGATTTGCCTTCTAGAACAGTAG and KRAS_A146T_R: CAGTGTTACTTACCTGTCTTGTCT.

Amplicon libraries were prepared from 1–3 ng plasma DNA as previously described by our group^10^. Briefly, target-specific primers were designed to amplify regions harboring the aforementioned variants and Illumina-specific adapters were added within a second PCR setup. Amplicon libraries were sequenced 150 bp paired-end on an Illumina NextSeq or MiSeq sequencer, obtaining a minimum of 200,000 reads per sample. Sequencing data were analyzed using an in-house pipeline and mutations were visualized using Integrative Genomics Viewer (IGV) (version 2.3.58).

### Statistical analysis

Statistical analysis was performed using R (version 4.0.2) and the ggpubr package (version 0.4.0). A *p*-value of < 0.05 was considered as statistically significant.

### Reporting summary

Further information on experimental design is available in the Nature Research Reporting Summary linked to this paper.

## Supplementary information

Supplementary Information Clean version

Reporting Summary Checklist

## Data Availability

The data generated and analyzed during this study are described in the following data record: 10.6084/m9.figshare.13078916^28^. The sequencing raw datasets have been deposited at the *European Genome-phenome Archive* (EGA; http://www.ebi.ac.uk/ega/) under study accession number https://identifiers.org/ega.study:EGAS00001004213^29^. This study contains the following four datasets: 1: sWGS of CRC patients (https://identifiers.org/ega.dataset:EGAD00001006101), 2: Mutation analysis data (AVENIO ctDNA Targeted Kit: https://identifiers.org/ega.dataset:EGAD00001006103, SiMSen-seq approach: https://identifiers.org/ega.dataset:EGAD00001006104, Deep sequencing: https://identifiers.org/ega.dataset:EGAD00001006105). Data access requests can be made to the appropriate Data Access Committee via the EGA landing page for each dataset. In addition to the sequencing data, the following data underlie the supplementary figures and tables of the related manuscript. Summary_file2.xlsx underlies Supplementary table 1 and Supplementary Figs. 8 and 10, and is available from the figshare repository at 10.6084/m9.figshare.13067348.v2^30^. 41586_2019_1272_MOESM2_ESM.xlsx underlies Supplementary fig. 11, and is available in the Supplementary Tables of Cristiano et al. (2019): 10.1038/s41586-019-1272-6^24^.

## References

[1] Ferlay J (2019). Estimating the global cancer incidence and mortality in 2018: GLOBOCAN sources and methods. Int. J. Cancer J. Int. du Cancer.

[2] Heitzer E, Haque IS, Roberts CES, Speicher MR (2019). Current and future perspectives of liquid biopsies in genomics-driven oncology. Nat. Rev. Genet..

[3] Diehl F (2008). Circulating mutant DNA to assess tumor dynamics. Nat. Med..

[4] Tie J (2015). Circulating tumor DNA as an early marker of therapeutic response in patients with metastatic colorectal cancer. Ann. Oncol..

[5] Garlan F (2017). Early evaluation of circulating tumor DNA as marker of therapeutic efficacy in metastatic colorectal cancer patients (PLACOL Study). Clin. Cancer Res..

[6] Deyme L, Barbolosi D, Gattacceca F (2019). Population pharmacokinetics of FOLFIRINOX: a review of studies and parameters. Cancer Chemother. Pharmacol..

[7] Siravegna G (2018). Radiologic and genomic evolution of individual metastases during HER2 blockade in colorectal cancer. Cancer Cell.

[8] Heitzer E (2013). Tumor-associated copy number changes in the circulation of patients with prostate cancer identified through whole-genome sequencing. Genome Med..

[9] Adalsteinsson VA (2017). Scalable whole-exome sequencing of cell-free DNA reveals high concordance with metastatic tumors. Nat. Commun..

[10] Heitzer E (2013). Complex tumor genomes inferred from single circulating tumor cells by array-CGH and next-generation sequencing. Cancer Res..

[11] Stahlberg A (2016). Simple, multiplexed, PCR-based barcoding of DNA enables sensitive mutation detection in liquid biopsies using sequencing. Nucleic Acids Res..

[12] Weber, S. et al. Technical evaluation of commercial mutation analysis platforms and reference materials for liquid biopsy profiling. *Cancers (Basel).*10.3390/cancers12061588 (2020). 10.3390/cancers12061588 PMC7352370 32560092

[13] El Messaoudi S (2016). Circulating DNA as a strong multimarker prognostic tool for metastatic colorectal cancer patient management care. Clin. Cancer Res..

[14] Bettegowda C (2014). Detection of circulating tumor DNA in early- and late-stage human malignancies. Sci. Transl. Med..

[15] Jahr S (2001). DNA fragments in the blood plasma of cancer patients: quantitations and evidence for their origin from apoptotic and necrotic cells. Cancer Res..

[16] Heitzer E, Auinger L, Speicher MR (2020). Cell-free DNA and apoptosis: how dead cells inform about the living. Trends Mol. Med..

[17] Lo YM (2010). Maternal plasma DNA sequencing reveals the genome-wide genetic and mutational profile of the fetus. Sci. Transl. Med..

[18] Jiang P (2015). Lengthening and shortening of plasma DNA in hepatocellular carcinoma patients. Proc. Natl Acad. Sci. USA.

[19] Mouliere F (2018). Enhanced detection of circulating tumor DNA by fragment size analysis. Sci. Transl. Med..

[20] Eisenhauer EA (2009). New response evaluation criteria in solid tumours: revised RECIST guideline (version 1.1). Eur. J. Cancer.

[21] Husain H (2017). Monitoring daily dynamics of early tumor response to targeted therapy by detecting circulating tumor DNA in urine. Clin. Cancer Res..

[22] Riediger AL (2016). Mutation analysis of circulating plasma DNA to determine response to EGFR tyrosine kinase inhibitor therapy of lung adenocarcinoma patients. Sci. Rep..

[23] Patsch K (2019). Monitoring dynamic cytotoxic chemotherapy response in castration-resistant prostate cancer using plasma cell-free DNA (cfDNA). BMC Res. Notes.

[24] Cristiano S (2019). Genome-wide cell-free DNA fragmentation in patients with cancer. Nature.

[25] Lampignano R (2020). Multicenter evaluation of circulating cell-free DNA extraction and downstream analyses for the development of standardized (Pre)analytical work flows. Clin. Chem..

[26] Ulz P (2016). Inferring expressed genes by whole-genome sequencing of plasma DNA. Nat. Genet..

[27] Stahlberg A (2017). Simple multiplexed PCR-based barcoding of DNA for ultrasensitive mutation detection by next-generation sequencing. Nat. Protoc..

[28] Moser, T. et al. Metadata record for the manuscript: on-treatment measurements of circulating tumor DNA during FOLFOX therapy in patients with colorectal cancer. *figshare.*10.6084/m6089.figshare.13078916 (2020). 10.1038/s41698-020-00134-3 PMC7666126 33299124

[29] European Genome-phenome Archive. https://identifiers.org/ega.study:EGAS00001004213 (2020).

[30] Moser, T. et al. Summary_file2. *figshare*. 10.6084/m6089.figshare.13067348.v2 (2020).

