# Supplementary material for: On-treatment measurements of circulating tumor DNA during FOLFOX therapy in patients with colorectal cancer
Source: NPJ Precis Oncol. 2020 Nov 13;4:30. doi: 10.1038/s41698-020-00134-3 (PMC7666126; doi:10.1038/s41698-020-00134-3)
Supplement: Supplementary file 1 — Supplementary Information Clean version [file 41698_2020_134_MOESM1_ESM.pdf]

On-treatment measurements of circulating tumor DNA during FOLFOX therapy in patients with colorectal cancer

Supplementary Information

Tina Moser[1,9], Julie Waldispuehl-Geigl[1,9], Jelena Belic[1,10], Sabrina Weber[1], Qing Zhou[1], Samantha O. Hasenleithner[1], Ricarda Graf[1], Jasmin Alia Terzic[2], Florian Posch[2], Heinz Sill[3], Sigurd Lax[4], Karl Kashofer[5], Gerald Hoefler[5], Helmut Schoellnast[6], Ellen Heitzer[1,7,8], Jochen B. Geigl[1], Thomas Bauernhofer[2,*], Michael R. Speicher[1,7,*]

[1] Institute of Human Genetics, Diagnostic and Research Center for Molecular Biomedicine, Medical University of Graz, Graz, Austria

[2] Department of Internal Medicine Graz, Division of Oncology, Medical University of Graz, Graz, Austria

[3] Department of Internal Medicine, Division of Hematology, Medical University of Graz, Graz, Austria

[4] Department of Pathology, General Hospital Graz II, Graz, Austria, and Johannes Kepler University Linz, Linz, Austria

[5] Institute of Pathology, Diagnostic and Research Center for Molecular Biomedicine, Medical University of Graz, Graz, Austria

[6] Department of Radiology, Division of General Radiology, Medical University of Graz, Graz, Austria

[7] BioTechMed-Graz, Graz, Austria

[8] Christian Doppler Laboratory for Liquid Biopsies for Early Detection of Cancer, Graz, Austria

[9] These authors contributed equally

[10] Present address: Cancer Research UK Cambridge Institute, University of Cambridge, CB2 0RE Cambridge, UK

*corresponding authors: thomas.bauernhofer@medunigraz.at; michael.speicher@medunigraz.at

Supplementary Note 1

FOLFOX (FOLinic acid (leucovorin), Fluorouracil (5-FU), OXaliplatin) treatment schedule (Fig. 1a)

- Oxaliplatin for 120 min on day 1;
- Leucovorin for 120 min on day 1;
- Fluorouracil (5-FU) as bolus transfusion on day 1 and 5-FU as continuous infusion over 46 hours;
- Repetition every 2 weeks for 4 cycles prior to radiological response evaluation.

Information on the pharmacokinetics of the individual substances
Data on FOLFOX pharmacokinetics is limited because the compounds were mostly studied in isolation and not as part of the FOLFOX combination. However, a recent study on the pharmacokinetics of FOLFIRINOX found no evidence that 5-FU and oxaliplatin may mutually impact their kinetics[13]. Furthermore, most studies report similar pharmacokinetics, which is usually characterized by a rapid increase in concentration in blood circulation after intravenous infusion followed by a swift decrease of concentration within minutes (5-FU) or 2-3 hours (oxaliplatin)[13].
Oxaliplatin: Oxaliplatin is an alkylating chemotherapy drug, which induces apoptosis of cancer cells through DNA damage, such as DNA lesions and inhibition of DNA as well as messenger RNA synthesis. Like other platinum compounds, its cytotoxicity is thought to result from inhibition of DNA synthesis in cells. In particular, oxaliplatin forms both inter- and intra-strand cross links in DNA, which prevent DNA replication and transcription, causing cell death. In both blood and plasma oxaliplatin may bind irreversibly to erythrocytes and plasma proteins so that platinum exists in free and bound fractions. While the bound fraction is inactive, only the free platinum fraction is capable of exerting anti-tumor action[13]. At the end of the two-hour infusion, 15% of the platinum administered is in the body's circulatory system, while the remaining 85% is rapidly distributed in the tissue or excreted with urine. Oxaliplatin undergoes extensive biotransformation in patients and no intact active substance is detectable in plasma ultrafiltrate at the end of a two-hour infusion. Platinum is predominantly excreted in urine, with clearance usually within 48 hours after administration.
5-fluorouracil (5-FU): 5-FU is an anti-metabolite, which acts on thymidylate synthase (TS) inhibition and thus prevents the formation of thymidine and consequently DNA synthesis. 5-FU acts principally as a thymidylate synthase (TS) inhibitor. Interrupting the action of this enzyme blocks synthesis of the pyrimidine thymidine, which is a nucleoside required for DNA replication. Thymidylate synthase methylates deoxyuridine monophosphate (dUMP) to form thymidine monophosphate (dTMP). Administration of 5-FU causes a scarcity in dTMP, so rapidly dividing cancerous cells undergo cell death via thymineless death. Therefore, 5-FU acts specifically on the S phase of the cell cycle[13]. Following intravenous administration, the half-life of 5-FU is very short and is estimated to be merely 8 to 14 minutes. After a single intravenously administered dose of fluorouracil, approximately 15% of the dose is excreted unchanged in the urine within 6 hours; of this, more than 90% is excreted within the first hours. The remainder is metabolized mainly in the liver, the vast majority of 5-FU is catabolized by dihydropyrimidine dehydrogenase (DPD) before it is eliminated in urine[13]. In general, there is a relationship between systemic plasma levels of 5-FU and treatment toxicity and efficacy. In order to achieve increased objective responses, high FU area under the curve (AUC) values have to be maintained. Depending on the patient status an $AUC_{0\text{-}8}$ of 20 to 25 $mg \cdot h \cdot L^{-1}$ is attempted to achieve in many studies[14]. Leucovorin can enhance

the binding of fluorouracil to an enzyme inside of cancer cells so that fluorouracil may stay in the cancer cell longer and exert its anti-cancer effect on the cells.

Supplementary Note 2

Patients and their detailed histories

Within this study, 13 CRC patients (DR1-DR13, mean age 61 years, range 45-71) were recruited at the Division of Oncology, Department of Internal Medicine, at the Medical University of Graz. The study was approved by Ethics Committee of the Medical University of Graz, Austria (approval number 26-288 ex 13/14) conducted according to the Declaration of Helsinki. Written informed consent was obtained from all patients.

We selected patients with metastasized CRC receiving FOLFOX therapy. Except for patients DR1, DR6 and DR13, all other patients received FOLFOX as first-line therapy. Two patients (DR5 and DR10) were excluded, as DR5 requested discontinuation from the study and DR10 did not meet the inclusion criteria. Clinicopathological features were retrieved from clinical and pathological records. Clinical characteristics are summarized in Supplementary Table 1 and detailed histories are provided in the following:

DR1:
Primary tumor: Mucinous adenocarcinoma of the cecum
Metastases: Local recurrence with infiltration of iliopsoas muscle
TNM: pT3 N0 MX
History: At the age of 67 years, this female was diagnosed with a mucinous adenocarcinoma, which infiltrated the subserous fatty tissue. The tumor was treated with a right-sided hemicolectomy with curative intent and an adjuvant chemotherapy with capecitabine (Xeloda®) for 5 months (8 cycles). About five years later, an infiltration of the right iliopsoas muscle was detected in a follow-up CT and was confirmed by biopsy and histopathology as relapse of the mucinous adenocarcinoma. This relapse was first treated with radiation, followed by treatment with capecitabine (Xeloda®), which had to be aborted after 1 cycle due to side effects (diarrhea). As a subsequent treatment, combination chemotherapy using the FOLFOX regimen was used and during the first three days of the second FOLFOX cycle, the blood for this study was collected.
Response: SD; Imaging: target lesion= musculus psoas; baseline SLD of target lesions= 112 mm; SLD after FOLFOX cycle=102 mm.

DR2:
Primary tumor: Adenocarcinoma of the hepatic flexure
Metastases: lung, abdominal wall metastases, lymph nodes, mesenterial metastases
TNM: pT4b N2a (4/23) M1
History: This male was diagnosed at 71 years of age with a colon adenocarcinoma in the right colic flexure. At the time of diagnosis, metastases at the abdominal wall and in the mesentery and lymph node infiltrations were noted. The patient underwent a palliative right-sided hemicolectomy and resection of the metastases in the abdominal wall and in the mesentery. About one month later, the first cycle of FOLFOX (without bevacizumab (Avastin®) due to prior port-á-cath implantation) was initiated. During this cycle. we obtained the blood samples for this study. The patient was treated for three months with FOLFOX plus bevacizumab and showed stable disease during the entire study period.
Response: SD; Imaging: due to the minimal tumor burden in the peritoneum, imaging was not sensitive enough to accurately quantify suitable target lesions.

DR3:
Primary tumor: Adenocarcinoma of the ascending colon
Metastases: liver
TNM: pTX
History: The diagnosis of an adenocarcinoma of the ascending colon and synchronous liver metastases were made in this male at the age of 53 years. A diagnostic biopsy was conducted to confirm the diagnosis. A comparison of abdominal CTs obtained two months earlier and immediately prior to our study revealed progression of the liver lesions with a size of 7 cm. FOLFOX treatment without bevacizumab ((Avastin®) due to prior port-á-cath implantation) was started about one month later, at which time we obtained the blood samples. The patient responded well to the therapy with regression of both the primary tumor and the liver metastasis. Five months after diagnosis and 11 cycles of FOLFOX plus bevacizumab, a palliative surgical resection of the primary tumor was performed.
Response: SD; Imaging: target lesions= liver x2), baseline SLD of target lesions= 130 mm; SLD after FOLFOX =114mm.

DR4:
Primary tumor: Adenocarcinoma of the rectosigmoid junction
Metastases: liver
TNM: pT4a N0 (0/20) M1
History: At the age of 57 years, two tumors of the rectum and sigmoid colon caused an ileus in this male, requiring emergency surgery. MR revealed a liver metastasis in segment VI (1.6x1.4 cm), and a second lesion in segment III (7 mm). Two months later, the first FOLFOX cycle without bevacizumab (Avastin®) due to prior port-á-cath implantation was administered during the first three days we collected the blood samples. The patient subsequently received cycles of FOLFOX with bevacizumab and the liver reference lesion SLD was 18 mm prior to therapy and 20 mm after four cycles. Four months after diagnosis, i.e. two months after the first FOLFOX treatment, metastases in segments III and VI were surgically removed with curative intent, which was followed by adjuvant FOLFOX treatment. After about four years of follow-up, the patient is still in complete remission and is doing well.
Response: SD: Imaging: target lesion= liver; baseline SLD of target lesions= 18 mm; SLD after 4 FOLFOX cycles= 20 mm.

DR6:
Primary tumors: Adenocarcinoma of the rectum; Breast cancer, diagnosed two and a half years prior to rectum adenocarcinoma
Metastases: lung, lymph nodes, abdominal
TNM: T2 N1a (1/14) MX
History: At the age of 55 years, invasive ductal carcinoma of the left breast was diagnosed in this female. A breast-conserving surgery was performed with curative intent (stage of disease: pT2 N2a (7/14) MX G2 R0; ER+ PR+ Her2neu: 2+). Subsequently, adjuvant chemotherapy (FEC/Taxotere) and postoperative radiation treatment were administered as well as adjuvant hormonal treatment using anastrozol (Arimidex®). Two and a half years later, i.e. at the age of 57 years, a rectum carcinoma was detected. A neoadjuvant chemo-radiation therapy with 5-FU was followed by surgical removal of the tumor with curative intent. Adjuvant therapy with capecitabine (Xeloda®) for a total of 6 cycles was offered post-operatively. However, 16 months later, metastases in the lung and in abdominal lymph nodes were noted in follow-up CT scans and confirmed with a diagnostic bronchoscopy, revealing metastasis of an adenocarcinoma of the rectum. Palliative

treatment with FOLFOX without bevacizumab ((Avastin®) due to port-á-cath implantation) was started and blood for our study was obtained during the first cycle. However, the follow-up imaging obtained two months later showed progressive disease and the patient died within two months thereafter.
Response: SD, according to the RECIST criteria, this patient had to be classified as SD although there was clear progression with clinical and radiological signs of an ileus. However, reference lymph nodes as measurable lesions according to RECIST criteria showed SD. Baseline SLD of target lesions= 54 mm; SLD after 4 FOLFOX cycles = 62 mm.

DR7:
Primary tumor: Rectum adenocarcinoma
Metastases: lung, liver
TNM: pT4a N2b (8/27) M1
History: This 45-year-old female was diagnosed with a rectum adenocarcinoma and synchronous lung and liver metastases. The rectum was resected and two months later, the patient received the first cycle of FOLFOX without bevacizumab ((Avastin®) due to prior port-á-cath implantation) during which we collected the blood samples. After the first 8 weeks of FOLFOX the patient showed stable disease and after 18 weeks partial response. Currently, i.e. three and a half years after diagnosis, the patient is being treated with monoclonal antibody-chemotherapy combinations (FOLFOX/bevacizumab (Avastin®)) and is responding well according to RECIST criteria.
Response: SD; Imaging: target lesions= lung x2 and liver; baseline SLD of target lesions= 32 mm; SLD after FOLFOX cycle =28 mm. Second follow-up imagining: PR; target lesions 18 weeks after 4 FOLFOX cycles = 19mm.

DR8:
Primary tumor: Adenocarcinoma of the sigmoid
Metastases: Carcinosis peritonei
TNM: pT3b N1a (1/12)
History: A diagnosis of an adenocarcinoma of the sigmoid was made in this male at the age of 65 years. He underwent surgery of this tumor with curative intent and received subsequently adjuvant chemotherapy with capecitabine (Xeloda®) for four months. However, 15 months later, a carcinosis peritonei was diagnosed and treatment with FOLFOX without bevacizumab ((Avastin®) due to prior port-á-cath implantation) was started. We collected the blood samples during the first three days of the first FOLFOX cycle. Restaging after four months was consistent with a partial response. However, at this time, the platinum-containing therapy had to be discontinued due to side effects. Maintenance treatment using leucovorin/fluorouracil (DeGramont) was initiated and follow-up imaging showed stable disease for 9 months.
Response: PR; Imaging: baseline SLD of target lesions= 47 mm; SLD after FOLFOX cycle=28 mm.

DR9:
Primary tumor: Adenocarcinoma of the cecum
Metastases: lung, lymph nodes (04/16)
History: At the age of 64 years, this female was diagnosed with a coecum adenocarcinoma, which was inoperable. The diagnosis was confirmed by a diagnostic colonoscopy. In addition, retroperitoneal lymph node metastases were detected. Palliative treatment with FOLFOX was started and the blood for this study was collected during the first cycle. Due to the *KRAS* wild-type status of the tumor panitumumab (Vectibi®) was added beginning with the second therapy cycle.

The patient remained stable for the next three months; however, four months after diagnosis, new lung metastases were noted and the patient died shortly thereafter.
Response: PR; Imaging: baseline SLD of target lesions= 34 mm; SLD after 4 FOLFOX cycles= 17 mm.

DR11:
Primary tumor: Adenocarcinoma of the rectum
Metastases: lung
TNM: pT3(p) N1a (1/22) pM1
History: A rectum adenocarcinoma with synchronous lung metastases was diagnosed in this male at the age of 66 years. The lesions in the lung were confirmed as metastases originating from the primary tumor of the rectum by biopsy and histopathology. The rectum and sigmoid colon were resected with palliative intent. One month later he received the first cycle of FOLFOX (without Bevacizumab (Avastin®) because of prior port-á-cath implantation), at which time the blood samples were collected. After three months of therapy, treatment was classified as "best response, stable disease"; and an 8-month break from therapy was made until progression of the lung metastases were noted.
Response: SD; Imaging: baseline SLD of target lesions= 28 mm; SLD after 4 FOLFOX cycles= 28 mm.

DR12:
Primary tumor: Mucinous adenocarcinoma of the sigma
Metastases: liver, carcinosis peritonei, pleural carcinosis
TNM: pT4a N2b (10/19) M1a
History: At the age of 63 years a mucinous adenocarcinoma of the sigma was diagnosed in this male patient. In addition, metastases in the liver and peritoneum were noted. Surgical treatment included an extended sigma resection and a resection of liver segment IV. One month later palliative treatment with FOLFOX without Bevacizumab (Avastin®) because of prior port-á-cath implantation was initiated and the blood samples were collected. However, the tumor progressed and the patient died four months after establishing the diagnosis.
Response: SD (even though this was a mixed response in the liver lesions, this does not exist in the RECIST criteria, so SD was designated); Imaging: target lesions= two each in the liver and in the lymph nodes; baseline SLD of target lesions= 71 mm; SLD after 4 FOLFOX cycles= 70 mm.

DR13:
Primary tumor: Adenocarcinoma of the rectum
Metastases: liver
TNM: pTX
History: An adenocarcinoma of the rectum was diagnosed in this male at 68 years of age. Synchronous liver metastases were noted. The diagnosis was confirmed by a diagnostic colonoscopy and biopsies. Initially, treatment consisted of FOLFIRI/Cetuximab (Erbitux®), which was switched to FOLFOX due to progressive disease six months after establishing the diagnosis. During the first FOLFOX cycle without Bevacizumab (Avastin®) due to rectal bleeding) the blood samples were obtained. The patient received altogether 3 cycles FOLFOX (May-Aug. 2017), which was then followed by radiation (Aug.-Sept. 2017).
Response: PD; Imaging: target lesions= liver; baseline SLD of target lesions= 140 mm; SLD after 3 FOLFOX cycles=172 mm.

Supplementary Note 3

Further details of the determination of tumor fractions in plasma DNA

Are daily variations of cfDNA a confounding factor?

Previously, it had been shown that cfDNA levels may fluctuate during the day[1]. Hence, we plotted our ctDNA levels according to the hours since treatment initiation, i.e. according to the different times during the day and we did not observe a significant decline during the day (Supplementary Fig. 8). Therefore, it is unlikely that cfDNA variations during daytime may have had a large impact on our observations.

Consistency between ichorCNA and mutation AFs

ichorCNA is an algorithm developed by Adalsteinsson and colleagues to quantify tumor content in cfDNA from 0.1x coverage whole-genome sequencing data[2]. We had previously described the plasma-Seq approach[3], which represents a shallow whole-genome sequencing technique for cfDNA and the output data are ideally suited for estimation of the tumor fraction using ichorCNA. Adalsteinsson and colleagues reported for their ichorCNA algorithm a lower detection limit of 3% tumor fraction in order to detect the presence of tumor with high sensitivity (0.95) and specificity (0.91)[2]. As determined with high-sensitivity approaches with a LOD of 0.1%, our cohort included several patients (DR1, DR2, DR4, DR7 and DR11), who had in all of their plasma samples a tumor fraction of less than 3%. As the tumor fraction in the plasma samples of these patients was below the resolution limit of ichorCNA, the tumor fraction was "undetected" based on ichorCNA (Supplementary Fig. 3; Fig. 2). In contrast, the respective mAFs were detectable with high-sensitivity approaches.

As shown in Figure 2, there may be differences between the ichorCNA determined tumor fraction and the corresponding estimates based on single mutations. For example, the ichorCNA estimated tumor fraction is compared to the mutation-based assays lower for plasma samples from patients DR9 and DR12, higher in cases DR6 and DR13, and about the same for DR3 and DR8. The most likely reason for different tumor fraction determinations is the localization of the respective mutation within the tumor genome, i.e. whether this region is gained/amplified or deleted. We analyzed this in detail previously [4]. In brief, if a mutation is located in a gained/amplified region, the corresponding tumor fraction estimate may be higher than the ichorCNA total tumor fraction determination and vice versa, if the mutation is in a deleted region it may be lower than the ichorCNA result. Furthermore, heterogeneity and preponderance of various clones may contribute to impact mAF levels of certain mutations. Hence, ichorCNA is less biased towards subclones or gene copy numbers and the total tumor fraction might differ from single mutation mAF estimates. However, it is very important that for the different measurements the course is very similar, if not even identical in terms of decreasing or increasing tumor fractions, such that an evaluation of the ctDNA AF course is possible regardless of the chosen method (see Fig. 2).

Consistency between AVENIO and SiMSen-seq

The AVENIO kit represents a commercial adaptation of cancer personalized profiling by deep sequencing (CAPP-Seq) [5,6], which has been applied in multiple publications. As such, both CAPP-seq and the AVENIO-kit represent established procedures in the field. In addition, we conducted extensive evaluations of this kit and our most recent efforts in this regard are reflected in a recent publication, which we produced as members of the CANCER-ID consortium. CANCER-ID is a public-private partnership supported by Europe's Innovative Medicines Initiative (IMI) consisting of 33 partners from 13 countries (www.cancer-id.eu). Within the framework of CANCER-ID, we

recently evaluated the use of commercially available reference materials designed for ctDNA testing and cfDNA for inter- and intra-assay as well as intra- and inter-laboratory comparisons. In addition to four other commercially available mutation assays, the performance of the AVENIO cfDNA targeted panel was assessed using 15ng of the Seraseq ctDNA reference material v2. The Seraseq ctDNA reference material is a full-process plasma-like material supporting the assessment of the entire workflow from extraction through the analysis. It includes 40 clinically relevant mutations across 28 genes at VAFs, i.e., 2%, 1%, 0.5%. 0.25%, 0.125%, and a wild-type (WT) sample and has become a standard for the evaluation of the performance of mutation detection assays.

In this comparison, the AVENIO panel was -as expected- one of the best performing assays (for details see ref.[7]), confirming the kit as a highly reliable and robust assay.

SiMSen-seq represents another established approach for ultrasensitive mutation detection[8,9]. However, the consistency between AVENIO and SiMSen-seq had not been previously tested. In order to test whether SiMSen-seq would represent a similar reliable approach as AVENIO, we used the Seraseq reference material as mentioned above and tested three mutations, i.e. *KRAS* G12D and *TP53* R273H and R248Q with SiMSen-seq.

For two mutations, i.e. *KRAS* G12D and *TP53* R273H, we obtained the expected high concordance between both assays (Supplementary Fig. 9). Surprisingly, for the third mutation, *TP53* R248Q, only SiMSen-seq yielded the expected allele frequencies from the Seracare reference, but the AVENIO assay underestimated the mAF (Supplementary Fig. 9). In this case the most likely explanation is that the AVENIO kit enriched more wild type fragments resulting in a reduced number of mutated fragments. In contrast, regions to be tested with SiMSen-seq are amplified by PCR, and for this mutation, this was apparently more efficient. Hence, SiMSen-seq even outperformed AVENIO for this particular mutation; however, as outlined above, due to our extensive experience and testing of the AVENIO kit[7], we have no doubt about the reliability of the AVENIO assay.

For further tests, we applied the AVENIO panel to the first plasma sample (T1) of DR13, which allowed us to identify a *TP53* mutation with an allele frequency of 8.7%. When we repeated the analysis with SiMSen-seq, we confirmed its presence of the *TP53* mutation with a similar mAF of 7.7%. In addition, we applied the AVENIO kit to plasma samples of one of the patients (DR2) with low ctDNA mAFs of less than 0.35% in all samples (Supplementary Fig. 3), in order to test whether the kit would allow identification of further mutations at such low ctDNA levels. AVENIO identified in the samples DR2_5 and DR2_6 a likely pathogenic *TP53* variant (p.Ser215Gly) with mAFs of 0.06% (DR2_5) and 0.30% (DR2_6). We applied SiMSen-seq to all nine plasma samples of DR2 and confirmed the presence of this variant in seven of the nine samples, each time again with a very low mAF of less than 0.3% (Supplementary Fig. 1).

Altogether, this data suggests a high consistency between AVENIO and SiMSen-seq. Importantly, to exclude any biases with mutation AF testing, we had added -as an orthogonal test- the SCNA-derived mAF as measured by the ichorCNA algorithm. As mentioned above, this orthogonal test showed in all instances with a mAF >3% the same course during our observation period, making it unlikely that any of our measurements will show major deviations or inaccuracies. Of note, in plasma samples with very low mAFs, stochastic effects alone may already cause different mAF estimates (for details see ref. [7]).

Potential impact of chemotherapy on plasma DNA levels

Chemotherapy may indeed represent an additional parameter that may have contributed to the increased plasma DNA levels. To address this question, we plotted the plasma DNA levels of healthy individuals next to the T1 values, i.e. before treatment, to demonstrate that the treatment

could not have caused the increased plasma DNA levels and furthermore, for additional comparisons T2-T8 (during therapy), and T9 (end of therapy). As shown in Supplementary Figure 10, the plasma DNA levels are already increased at T1, indicating that this increase is not due to chemotherapy.

Repeated analyses with an age-matched healthy control group

Our healthy control group consisted of individuals in the age range of 20 to 29 years and are therefore younger than our cancer cohort. In a previously published meta-analysis, which included a total of 2176 cancer patients and 892 healthy controls (from 8 studies), the distribution between age and plasma DNA concentration was compared and it was concluded that this age difference is not decisive[10].

To further exclude that the age difference between healthy controls and patients did not affect our conclusions, we conducted the following analysis: We used data of the healthy controls from the study by Cristiano et al.[11]. In this study, a total of 245 healthy individuals were analyzed (age group: 34-80 years, mean: 57 years). For our analysis, we included people in the age group 45-71 years (therefore 208 individuals were included). cfDNA had been isolated with the QIAGEN Circulating Nucleic Acids Kit. Since no method deviations were documented, we assume that the samples were isolated according to the same protocol we used, i.e. with the inclusion of carrier RNA.

We then repeated our analyses and found that also with the age-matched samples from the Cristiano et al. study, that the differences between healthy controls and patients were highly significant (Supplementary Fig. 11).

Supplementary Note 4

Evaluation according to RECIST criteria

Responses and tumor progression were assessed according to standard RECIST 1.1 criteria [12], which are considered to be the current gold standard for the assessment of disease bulk and treatment response definition, image-based response evaluation. In brief, target lesions representative of involved organs were selected on the basis of their size (lesions with the longest diameter) and whether they allowed reproducible repeated measurements. Up to a maximum of two lesions per organ and five lesions in total were identified as target lesions and recorded and a sum of the longest diameter (SLD) for all target lesions is calculated. A more than 30% but less than 100% decrease in the sum of the SLD of target lesions corresponds to PR, while a more than 20% increase in the sum of target lesion SLD corresponds to PD.

Supplementary Table

Supplementary Table 1. Summary of patient clinical characteristics

Patient ID	Gender	Age at diagnosis	Days between plasma and diagnosis	Primary tumor location	Histotype	Tumor Stage	Mutational status of primary tumor	Mutations identified using the AVENIO ctDNA Targeted Panel	Metastatic sites at the time of first blood draw
DR1	F	66.9	2132	Cecum	MA	pT3 N0 Mx	*KRAS* p.Ala146Thr	NA	Psoas muscle
DR2	M	70.7	58	Hepatic flexure	AD	pT4b N2a M1	*KRAS* p.Gly12Asp	*KRAS* p.Gly12Asp, *TP53* p.Ser215Gly	Lung, Lymph nodes (resection of adominal wall and mesenteric metastases)
DR3	M	52.7	35	Ascending colon	AD	pTx	*KRAS* p.Gly12Asp	NA	Liver
DR4	M	57.1	78	Rectosigmoid junction	AD	pT4a N0 M1	*KRAS* p.Lys117Asn (liver metastases)	*APC* p.Arg232*, *APC* p.Arg283*, *KIT* p.?, *KRAS* p.Lys117Asn, *TP53* p.Arg175His	Liver
DR6	F	57.5	831	Rectum	AD	pT2 N1a Mx	*KRAS* p.Gly13Asp	NA	Lung, Lymph nodes, Adrenal gland
DR7	F	45.3	70	Rectum	AD	pT4a N2b M1	*KRAS* p.Gly12Asp	NA	Liver, Lung
DR8	M	65.7	496	Sigmoid	AD	pT3b N1a	*KRAS* p.Gly12Val	NA	Peritoneum (carcinosis peritonei)
DR9	F	64.2	29	Cecum	AD	NA	*BRAF* p.Val600Glu *TP53* p.Arg337Cys	*BRAF* p.Val600Glu, *TP53* p.Arg337Cys	Lymph nodes
DR11	M	65.6	43	Rectum	AD	pT3 N1a M1	*KRAS* p.Gly12Asp	ND	Lung
DR12	M	63.2	82	Sigmoid	MA	pT4a N2b M1a	*KRAS* p.Ala146Thr	NA	Liver, Lymph nodes, Peritoneum (carcinosis peritonei)
DR13	M	68.7	191	Rectum	AD	pTx	WT	*TP53* p.Gly245Ser	Liver

F: female; M: male; MA: mucinous adenocarcinoma; AD: adenocarcinoma; NA: not applied; ND: not detected

Supplementary Figures

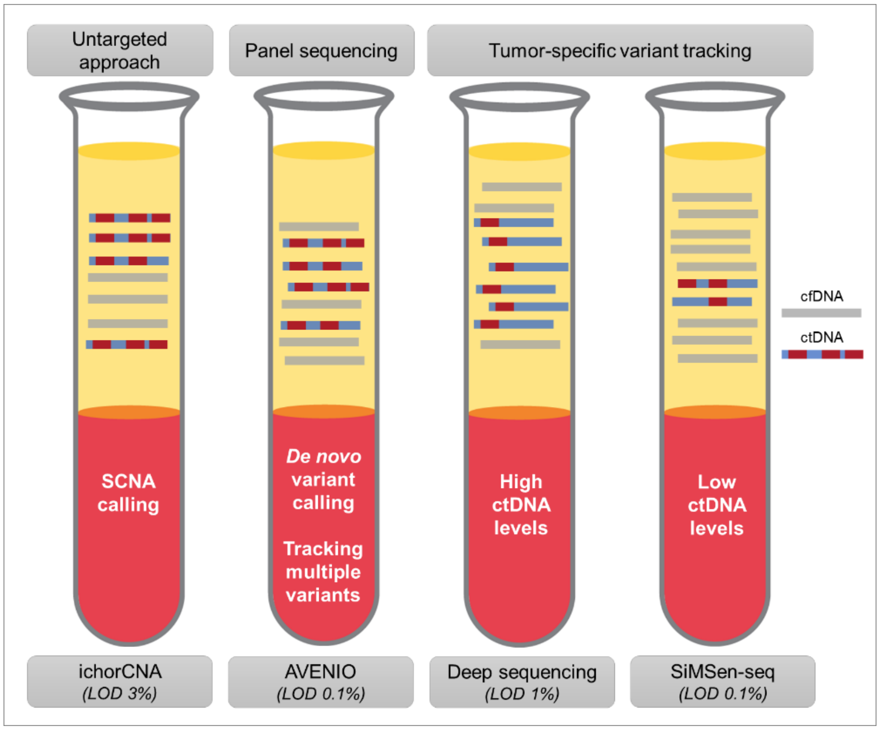

Supplementary Figure 1. Identification of tumor-specific alterations in plasma of CRC patients.

All plasma samples were analyzed using an untargeted sequencing approach and the respective tumor fractions were determined with the ichorCNA algorithm (LOD 3%)[2]. The AVENIO Targeted kit[7] (LOD 0.1%) was applied for a *de novo* mutation analysis in some plasma samples from DR2, DR4, DR9, and DR13. The same kit was used for longitudinal analysis of plasma samples from DR4 and DR9. In plasma samples (DR3, DR8, DR12) with high ctDNA levels, we performed deep sequencing (LOD 1%)[15] to track dynamic changes of the respective tumor-specific *KRAS* mutations. To monitor known *KRAS* mutations and/or variants identified using the AVENIO kit in patients (DR1, DR2, DR6, DR7, DR11, DR13) with low ctDNA levels, we employed SiMSen-seq (LOD 0.1%)[8,9].

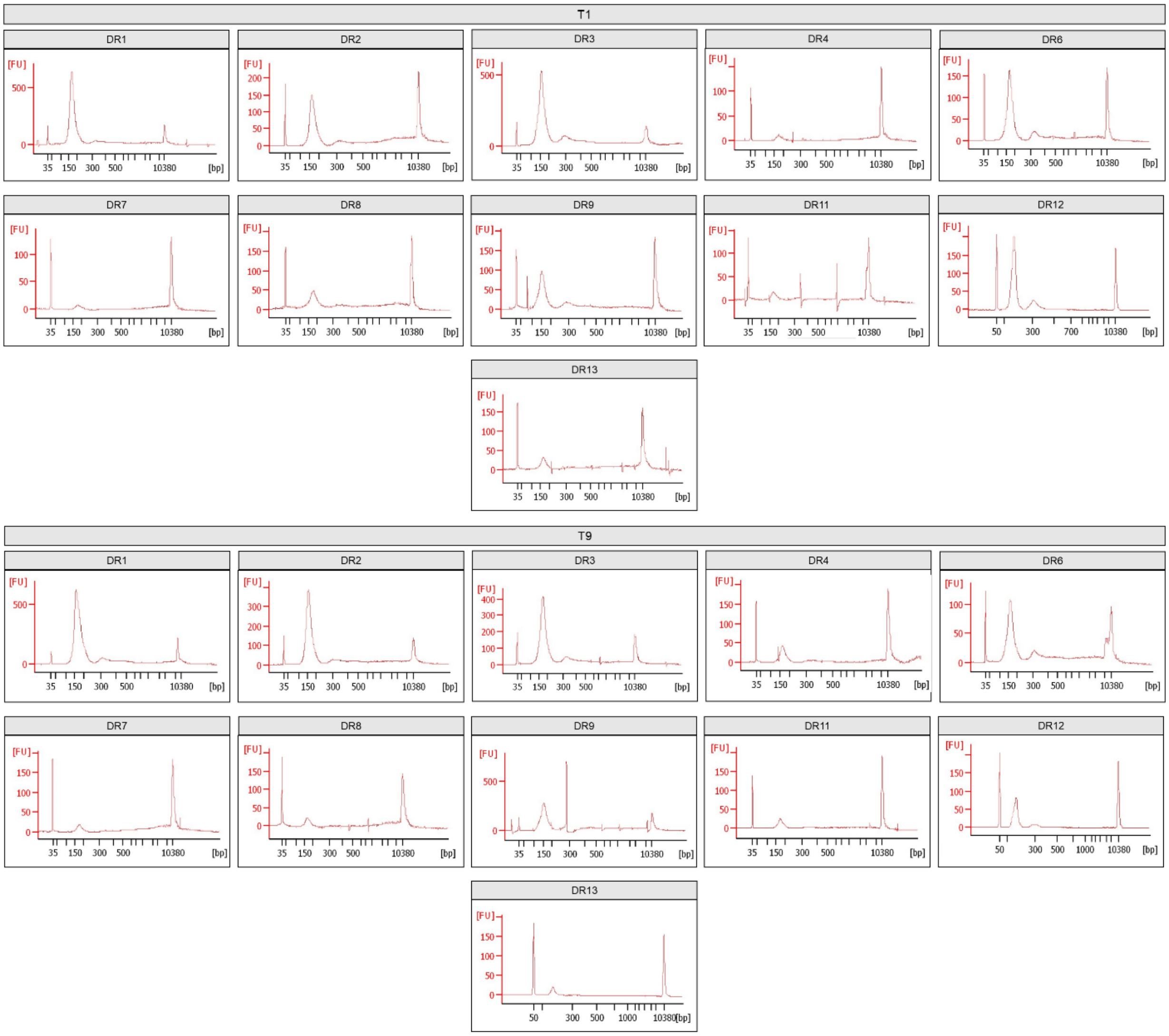

Supplementary Figure 2. Bioanalyzer traces from all patients at T1 and T9.

The bioanalyzer plots from all patients are comparable. The plots of DR12 indicate that the exceptionally increased plasma DNA concentrations of this patient were not caused by an admixture of high-molecular weight DNA derived from blood cells.

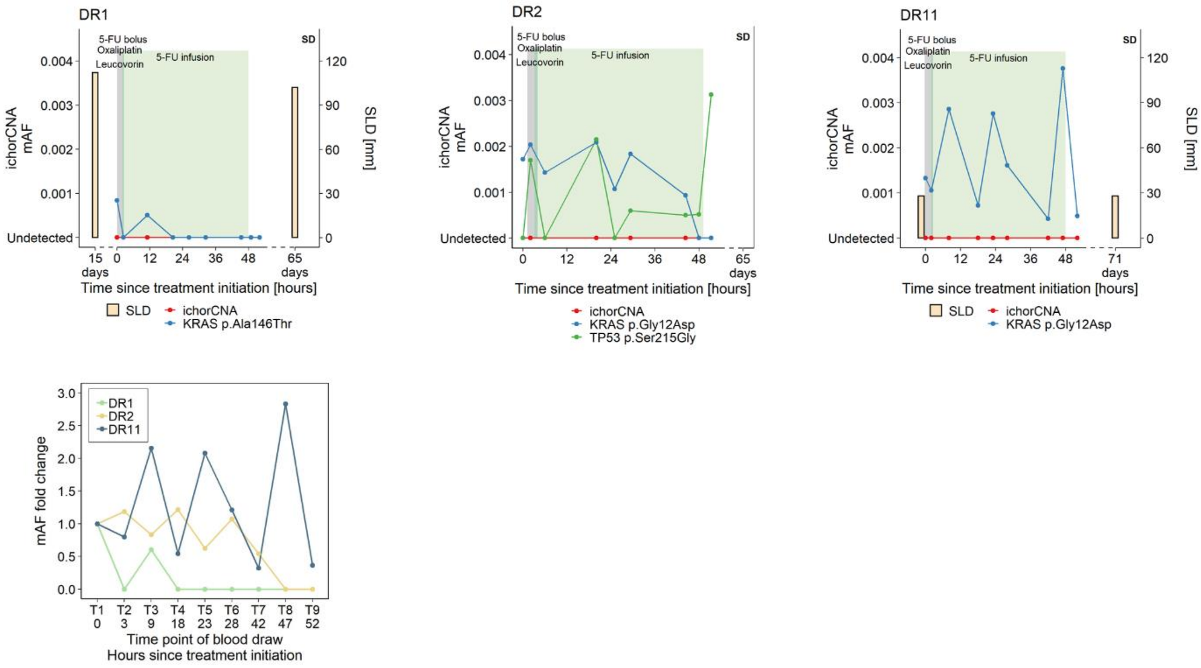

Supplementary Figure 3. ctDNA levels in plasma from patients DR1, DR2 and DR11. Summary of three patients (DR1, DR2, DR11) with mutant allele frequencies (mAFs) of less than 0.4% in all analyses; in each panel, the tumor fraction as established by ichorCNA is displayed in red, the respective *KRAS* mutations in blue and in case of DR2 the *TP53* mutation in green. Before and after the FOLFOX cycle, the sum of longest diameters (SLD) for the target lesions is displayed, with the exception of DR2. Plasma ctDNA mAF fold changes are displayed in the lower panel. Due to the low mAFs in the plasma of these patients, minor mAF fluctuations may result in relatively large appearing fold changes.

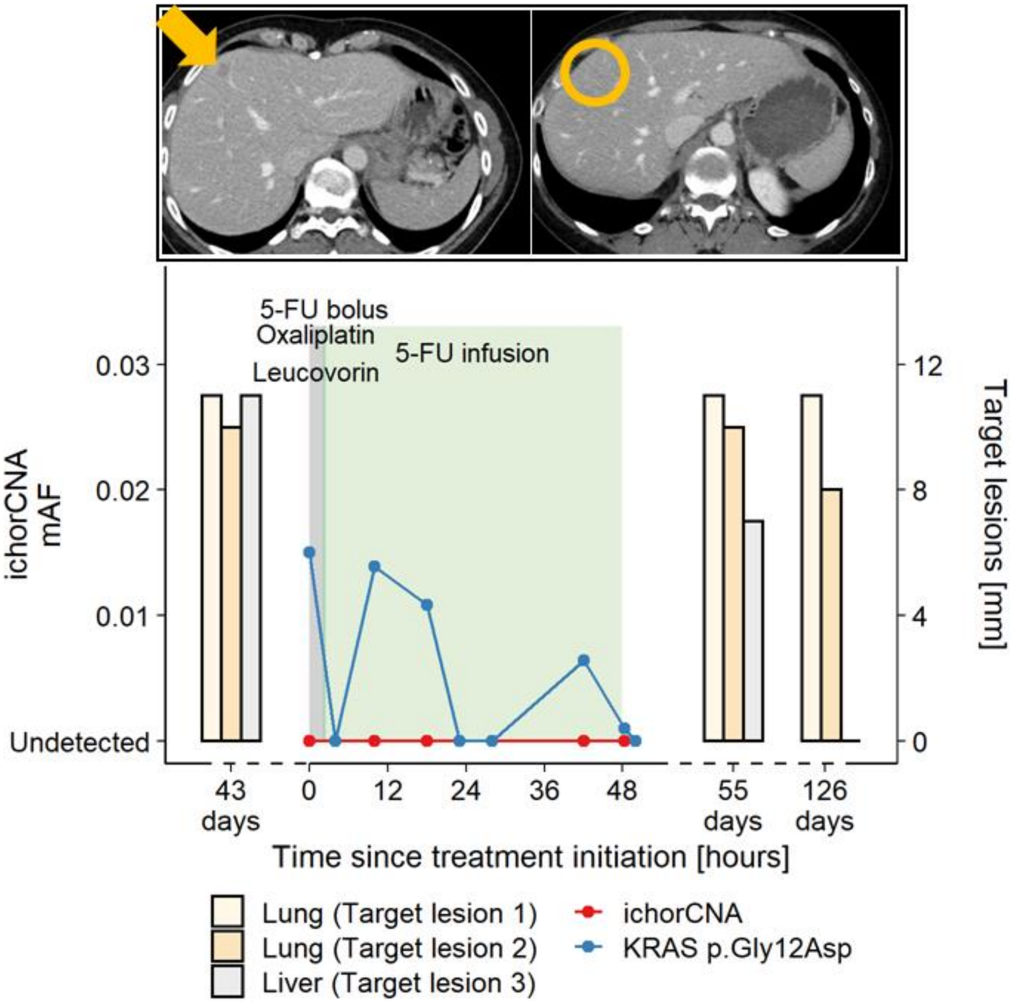

Supplementary Figure 4. Summary of disease response over time for patient DR7 with CT scans.

Section of Fig. 2a illustrating the *KRAS* mutation allele frequency, the ichorCNA tumor fraction and the longest diameter of all three target lesions of patient DR7 together with CT scans from the liver taken before and after the treatment cycle. Baseline CT scan showed a metastatic lesion in the right liver lobe (arrow). Follow-up imaging, which was taken 18 weeks after treatment, revealed that the previous liver lesion had completely disappeared (circle).

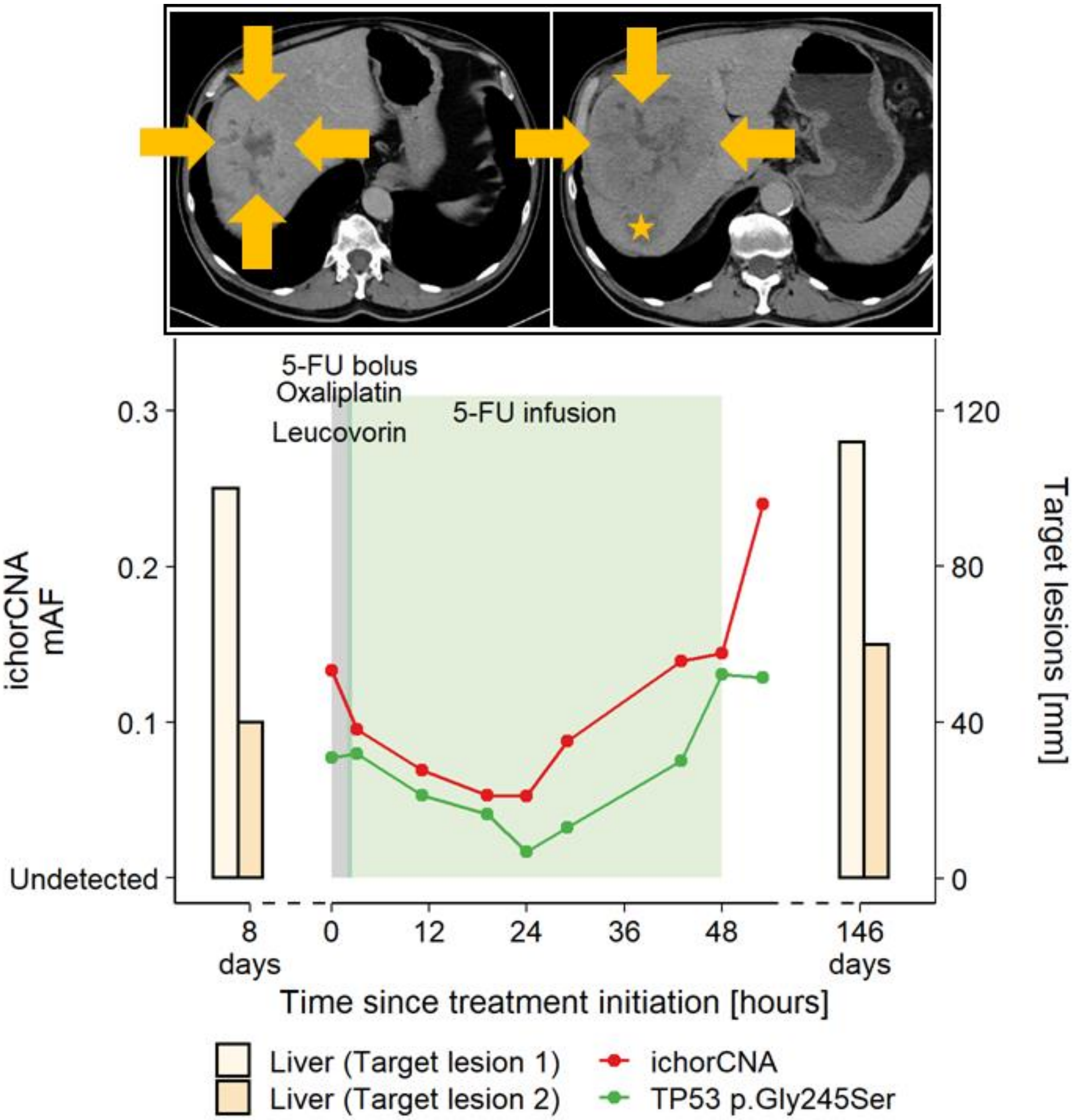

Supplementary Figure 5. Summary of disease response over time for patient DR13 with CT scans.

Section of Fig. 2c showing the mAF of a *TP53* mutation, the tumor fraction as determined by ichorCNA and the size of two target lesions along with CT scans of the liver demonstrating the progressive disease for case DR13. CT scan, taken before treatment, demonstrated a metastatic lesion in the right liver lobe (arrows) with central necrosis. Follow-up CT imaging 21 weeks after treatment initiation showed a clear enlargement of the lesion (arrows) with a new satellite lesion near the target lesion (asterisk).

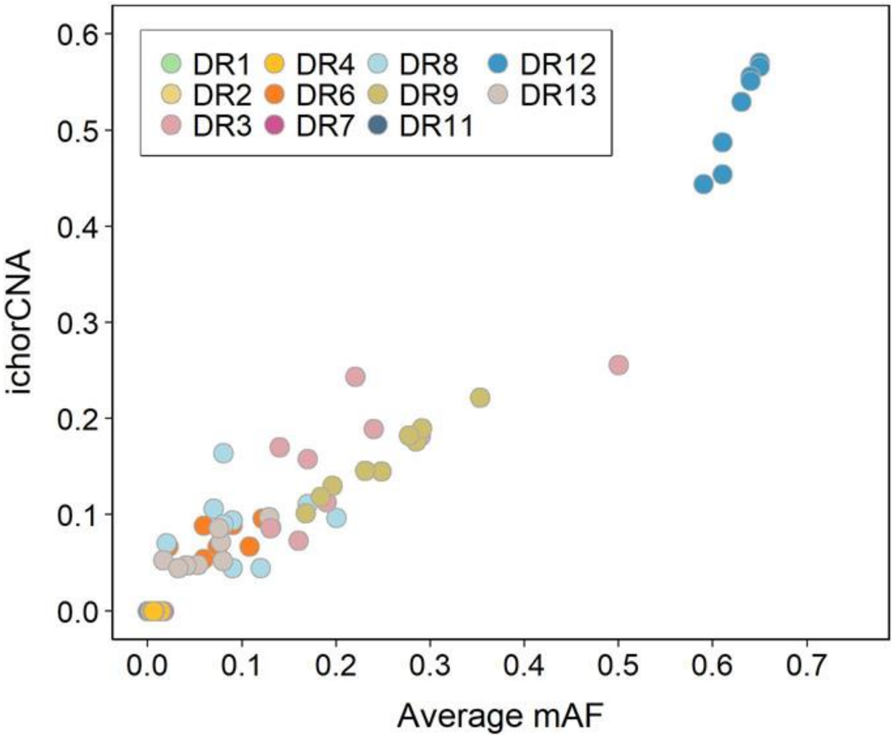

Supplementary Figure 6. Concordance between average mutant mAF and SCNA-based tumor fraction.

Concordance between average mAF and ichorCNA tumor fraction (Lin's concordance correlation coefficient CCC=0.94 and Pearson's correlation coefficient R=0.98, $p < 2.2*10^{-16}$).

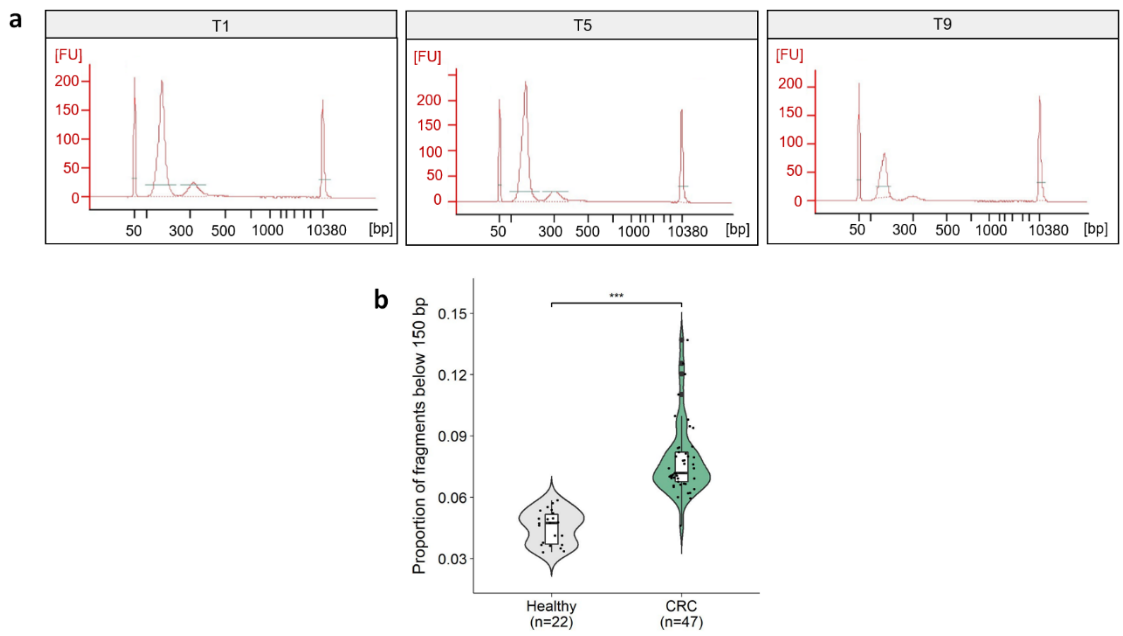

Supplementary Figure 7. Size profiles of cfDNA.

a Automated electrophoreses using the Bioanalyzer instrument for plasma samples taken at T1, T5 and T9 from patient DR12. **b** Proportion of cfDNA fragments below 150 bp determined by paired-end sequencing of plasma samples from healthy controls and CRC patients (n= 47 plasma samples; Wilcoxon, $p < 0.0001$).

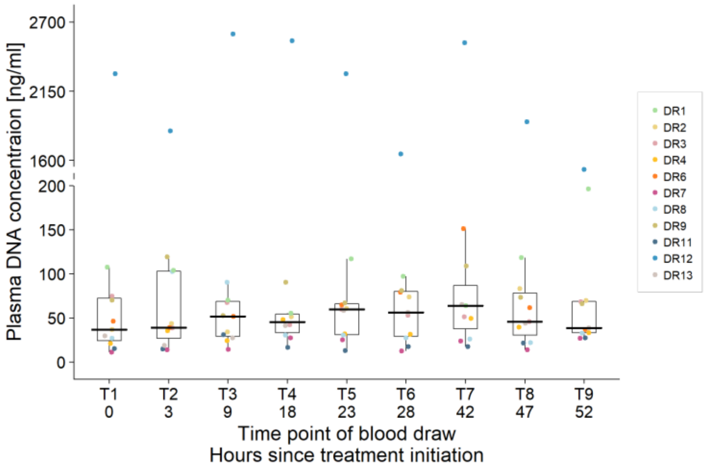

Supplementary Figure 8: Concentration of cfDNA during FOLFOX administration.

cfDNA levels of the patients are plotted according to hours since treatment initiation (T1-T9). The cfDNA concentrations are expressed in ng/ml of plasma. Boxplot indicates the median, interquartile range and the minimum and maximum.

Mutation	Expected	*SiMSen-seq*	*AVENIO*
KRAS p.G12D	VAF 2%	1,53	1,80
KRAS p.G12D	VAF 1%	1,03	1,10
KRAS p.G12D	VAF 0.5%	0,47	0,60
KRAS p.G12D	VAF 0.25%	0,24	0,30
KRAS p.G12D	VAF 0.125%	0,21	0,00
Mutation	Expected	*SiMSen-seq*	*AVENIO*
TP53 p.R273H	VAF 2%	1,75	2,20
TP53 p.R273H	VAF 1%	0,76	1,00
TP53 p.R273H	VAF 0.5%	0,16	0,00
TP53 p.R273H	VAF 0.25%	0,24	0,30
TP53 p.R273H	VAF 0.125%	0,19	0,00
Mutation	Expected	*SiMSen-seq*	*AVENIO*
TP53 p.R248Q	VAF 2%	2,48	0,50
TP53 p.R248Q	VAF 1%	1,08	0,20
TP53 p.R248Q	VAF 0.5%	0,50	0,20
TP53 p.R248Q	VAF 0.25%	0,16	0,00
TP53 p.R248Q	VAF 0.125%	0,17	0,00

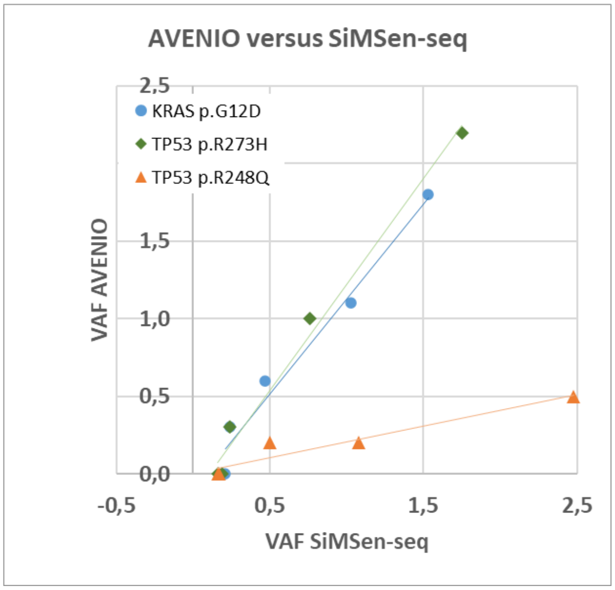

Supplementary Figure 9: Comparison AVENIO and SiMSen-seq.

Seraseq ctDNA V2 reference materials contain *KRAS* p.G12D, *TP53* p.R273H, and *TP53* p.R248Q mutations at different variant allele frequencies (VAF; 2%, 1%, 0.5%, 0.25%, 0.125%), as shown in the expected row (left panel). The VAFs of different variants (displayed in different colors) detected by SiMSen-seq and AVENIO (right panel) are shown on the x-axis and y-axis, respectively.

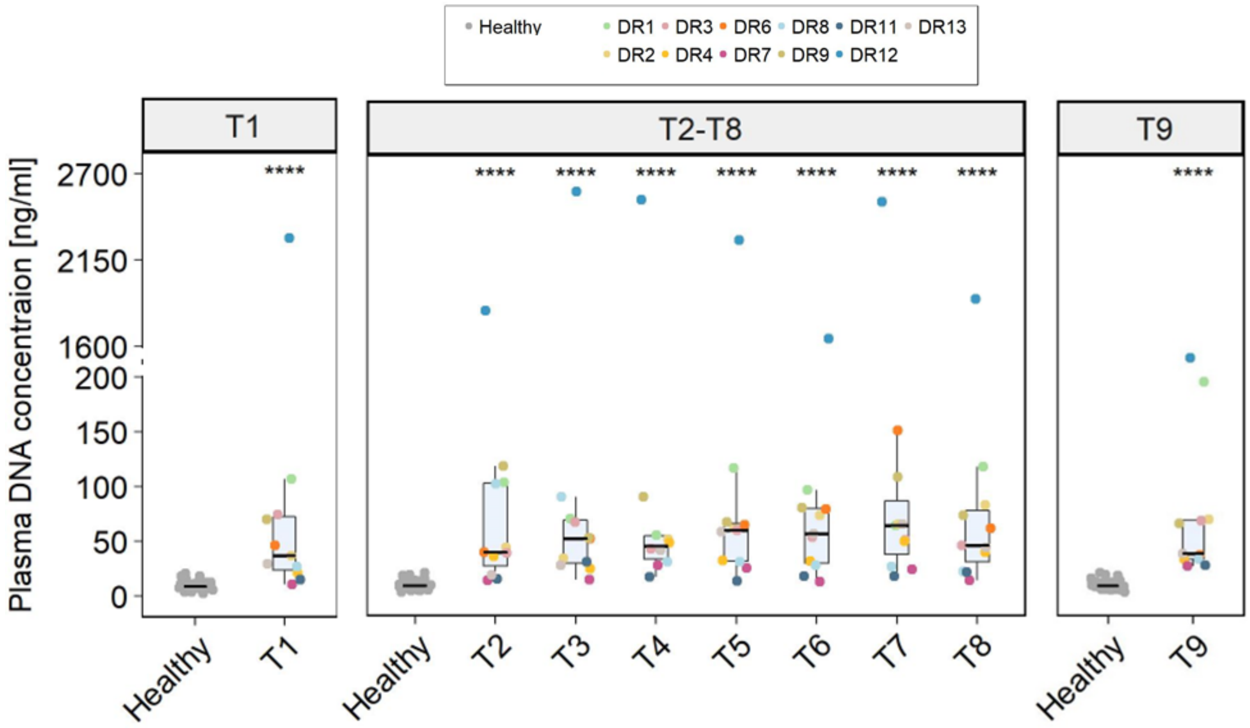

Supplementary Figure 10: cfDNA concentrations of healthy controls and CRC individuals. Plasma DNA quantities of healthy controls (n=60) and the patients subdivided according T1 (prior to treatment), T2-T8 (during treatment), and T9 (after treatment) (Mann-Whitney's *U* test; ****p <0.0001). All boxplots indicate the minimum and maximum value and median (center), and the interquartile range is shown by box and whiskers.

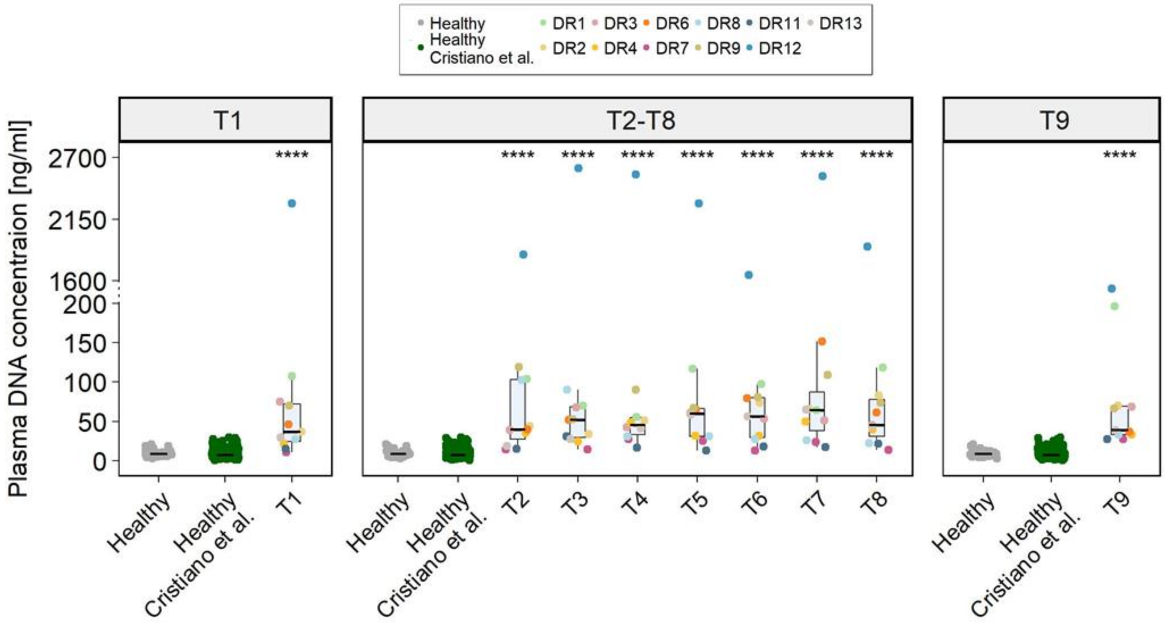

Supplementary Figure 11: Same plot as in Supplementary Figure 10 but with the addition of age-matched healthy controls from the study by Cristiano and colleagues [11].

References

1 Madsen, A. T., Hojbjerg, J. A., Sorensen, B. S. & Winther-Larsen, A. Day-to-day and within-day biological variation of cell-free DNA. *EBioMedicine* **49**, 284-290, doi:10.1016/j.ebiom.2019.10.008 (2019).

2 Adalsteinsson, V. A. *et al.* Scalable whole-exome sequencing of cell-free DNA reveals high concordance with metastatic tumors. *Nature communications* **8**, 1324, doi:10.1038/s41467-017-00965-y (2017).

3 Heitzer, E. *et al.* Tumor-associated copy number changes in the circulation of patients with prostate cancer identified through whole-genome sequencing. *Genome Med* **5**, 30, doi:10.1186/gm434 (2013).

4 Ulz, P., Heitzer, E., Geigl, J. B. & Speicher, M. R. Patient monitoring through liquid biopsies using circulating tumor DNA. *International journal of cancer. Journal international du cancer* **141**, 887-896, doi:10.1002/ijc.30759 (2017).

5 Newman, A. M. *et al.* An ultrasensitive method for quantitating circulating tumor DNA with broad patient coverage. *Nature medicine* **20**, 548-554, doi:10.1038/nm.3519 (2014).

6 Newman, A. M. *et al.* Integrated digital error suppression for improved detection of circulating tumor DNA. *Nature biotechnology* **34**, 547-555, doi:10.1038/nbt.3520 (2016).

7 Weber, S. *et al.* Technical Evaluation of Commercial Mutation Analysis Platforms and Reference Materials for Liquid Biopsy Profiling. *Cancers (Basel)* **12**, doi:10.3390/cancers12061588 (2020).

8 Stahlberg, A. *et al.* Simple multiplexed PCR-based barcoding of DNA for ultrasensitive mutation detection by next-generation sequencing. *Nature protocols* **12**, 664-682, doi:10.1038/nprot.2017.006 (2017).

9 Stahlberg, A. *et al.* Simple, multiplexed, PCR-based barcoding of DNA enables sensitive mutation detection in liquid biopsies using sequencing. *Nucleic acids research* **44**, e105, doi:10.1093/nar/gkw224 (2016).

10 van der Pol, Y. & Mouliere, F. Toward the Early Detection of Cancer by Decoding the Epigenetic and Environmental Fingerprints of Cell-Free DNA. *Cancer cell* **36**, 350-368, doi:10.1016/j.ccell.2019.09.003 (2019).

11 Cristiano, S. *et al.* Genome-wide cell-free DNA fragmentation in patients with cancer. *Nature* **570**, 385-389, doi:10.1038/s41586-019-1272-6 (2019).

12 Eisenhauer, E. A. *et al.* New response evaluation criteria in solid tumours: revised RECIST guideline (version 1.1). *European journal of cancer* **45**, 228-247, doi:10.1016/j.ejca.2008.10.026 (2009).

13 Deyme, L., Barbolosi, D. & Gattacceca, F. Population pharmacokinetics of FOLFIRINOX: a review of studies and parameters. *Cancer chemotherapy and pharmacology* **83**, 27-42, doi:10.1007/s00280-018-3722-5 (2019).

14 Gamelin, E. *et al.* Individual fluorouracil dose adjustment based on pharmacokinetic follow-up compared with conventional dosage: results of a multicenter randomized trial of patients with metastatic colorectal cancer. *Journal of clinical oncology : official journal of the American Society of Clinical Oncology* **26**, 2099-2105, doi:10.1200/JCO.2007.13.3934 (2008).

15 Heitzer, E. *et al.* Complex tumor genomes inferred from single circulating tumor cells by array-CGH and next-generation sequencing. *Cancer research* **73**, 2965-2975, doi:10.1158/0008-5472.CAN-12-4140 (2013).
